# In Situ Fabrication of Mn-Doped NiMoO_4_ Rod-like Arrays as High Performance OER Electrocatalyst

**DOI:** 10.3390/nano13050827

**Published:** 2023-02-23

**Authors:** Shiming Yang, Santosh K. Tiwari, Zhiqi Zhu, Dehua Cao, Huan He, Yu Chen, Kunyapat Thummavichai, Nannan Wang, Mingjie Jiang, Yanqiu Zhu

**Affiliations:** 1 Key Laboratory of Disaster Prevention and Structural Safety of Ministry of Education, Guangxi Key Laboratory of Disaster Prevention and Engineering Safety, State Key Laboratory of Featured Metal Materials and Life-Cycle Safety for Composite Structures, School of Resources, Environment and Materials, Guangxi University, Nanning 530004, China; 2 College of Engineering, Mathematics and Physical Sciences, University of Exeter, Exeter EX4 4QF, UK; 3 Department of Chemistry, NMAM Institute of Technology, Nitte (Deemed to be University), Nitte 547110, Karnataka, India; 4 Department of Mathematics, Physics and Electrical Engineering, Faculty of Engineering and Environment, Northumbria University, Newcastle-upon-Tyne NE1 8ST, UK

**Keywords:** OER, Mn-doped, transition metal electrocatalyst, nanosheet

## Abstract

The slow kinetics of the oxygen evolution reaction (OER) is one of the significant reasons limiting the development of electrochemical hydrolysis. Doping metallic elements and building layered structures have been considered effective strategies for improving the electrocatalytic performance of the materials. Herein, we report flower-like nanosheet arrays of Mn-doped-NiMoO_4_/NF (where NF is nickel foam) on nickel foam by a two-step hydrothermal method and a one-step calcination method. The doping manganese metal ion not only modulated the morphologies of the nickel nanosheet but also altered the electronic structure of the nickel center, which could be the result of superior electrocatalytic performance. The Mn-doped-NiMoO_4_/NF electrocatalysts obtained at the optimum reaction time and the optimum Mn doping showed excellent OER activity, requiring overpotentials of 236 mV and 309 mV to drive 10 mA cm^−2^ (62 mV lower than the pure NiMoO_4_/NF) and 50 mA cm^−2^ current densities, respectively. Furthermore, the high catalytic activity was maintained after continuous operation at a current density of 10 mA cm^−2^ of 76 h in 1 M KOH. This work provides a new method to construct a high-efficiency, low-cost, stable transition metal electrocatalyst for OER electrocatalysts by using a heteroatom doping strategy.

## 1. Introduction

The increasingly serious environmental pollution and the excessive consumption of fossil fuels have prompted people to start looking for efficient and green energy [1]. Hydrogen, due to its high combustion value, non-polluting, and abundant source, is seen as a highly efficient and clean energy alternative to traditional fossil energy sources and has attracted increasing scientific attention [2]. Electrochemical hydrolysis is an up-and-coming technology for sustainable hydrogen production with zero carbon emissions [3]. Two half-reactions are involved in electrochemical hydrolysis: the hydrogen evolution reaction (HER) and the oxygen evolution reaction (OER). The slow kinetics of the four-electron transfer for OER requires a larger overpotential to drive the reaction [4,5]. Electrocatalysts can effectively reduce the reaction energy barrier and accelerate the charge transfer rate. Thus far [6], RuO_2_ and IrO_2_ are known as efficient catalysts for HER and OER, respectively, however, the low stability, high cost, and scarcity of resources of these noble metal catalysts remain and limit their widespread application [7]. Therefore, designing a highly stable, low-cost, and widely available OER catalyst is imperative.

In recent years, transition metal oxide materials have been widely used as OER electrocatalysts due to their low cost, abundant resources, and considerable stability under alkaline conditions [8,9,10]. Meanwhile, bimetallic oxides exhibit better electrical conductivity and electrochemical properties due to the interactions between their internal metal cations such as NiCo_2_O_4_ [11], NiFe_2_O_4_ [12], NiWO_4_ [13], CoMoO_4_ [14], and NiMoO_4_ [15,16,17,18,19]. Notably, NiMoO_4_ micron rods with high aspect ratios have been reported to have a large specific surface area and are easy to synthesize, and Ni ions can easily convert to NiOOH under alkaline conditions, which favor facilitating the OER reaction [20]. According to previous reports, anisotropic MoO_4_^2−^ can modulate the structure and thus expose more active sites [21]. However, NiMoO_4_, as an electrocatalyst, suffers from a tendency to agglomerate, the need for additional binder assistance, low electrical conductivity, and lack of active sites [22].

Metal ion doping (such as Mn, Fe) or hybridized with graphene oxide could help to enhance the density of the active site and improve the electrical conductivity, thus reducing the energy barrier for water adsorption and dissociation [19,23,24,25,26]. It is worth noting that the doping of Mn elements tends to increase the conductivity and activity of transition metal-based electrocatalysts and improve their OER performance [27,28]. On the other hand, the doping of Mn ions can effectively improve the electron transfer efficiency and enhance the OER reaction process [29,30]. For example, Xu et al. reported Mn-doped Ni_2_P/Ni_5_P_4_ nanorods that grown on Ni foam that only required an overpotential of 230 mV (versus RHE) at a current density of 10 mA cm^−2^ [31]. Duan et al. prepared Fe, Mn-Ni_3_S_2_/NF by doping with Fe and Mn elements and an overpotential of only 216 mV was required to drive a current density of 30 mA cm^−2^ and it maintained the desirable stability for 45 h [32]. In situ growth of the active material on a conductive substrate is a preferred strategy over conventional powder catalysts [33] as it allows the catalyst to be tightly bonded to the substrate without the need for additional binders, improving the electron transfer efficiency, accelerating the diffusion of bubbles, and is also suitable for long time testing [34].

In order to increase the surface area of the electrocatalyst and to expose more active sites, the different morphologies of the electrocatalyst play an important role. Gong et al. prepared four different morphologies of Mn_2_-CNN/NF by a two-step hydrothermal and one-step calcination method and found that the flower-like nanosheet array structure of Mn_2_-CNN/NF exhibited excellent electrochemical properties [35], providing us with an idea to prepare electrocatalysts with different morphologies. Yu et al. developed porous nanosheet Mn-NiCo_2_S_4_/NF catalysts and exhibited good catalytic performance and stability, which proved that the doping of metal atoms allows the electronic structure and balance between overpotential and current to be modulated [36]. The choice of a suitable substrate material reduces the ineffective volume created by the binder, enhances the interaction between the electrocatalyst and the conducting substrate, and reinforces stability in electrolyte solutions at high current densities, making the 3D porous Ni foam a good choice [37,38].

Herein, we successfully prepared Mn-doped-NiMoO_4_/NF nanosheet arrays on Ni foam substrates and performed a series of electrochemical tests. As for the results, the electrocatalysts required overpotentials of only 236 mV and 309 mV to drive current densities of 10 mA cm^−2^ and 50 mA cm^−2^, respectively. Furthermore, it remained stable for at least 76 h under alkaline test conditions at 1 M KOH. In addition, Mn-doped-NiMoO_4_/NF (25) had a smaller Tafel slope of 91.89 mV dec^−1^. The experimental results suggest that the excellent electrocatalytic performance of Mn-doped-NiMoO_4_/NF (25) may be attributed to the unique vertical array structure of the nanosheets formed by the Mn elemental doping, which modulates the NiMoO_4_/NF morphology and provides more electrochemically active sites and channels for electrolyte ion diffusion as well as good electrical conductivity.

## 2. Experimental Section

### 2.1. Materials

All laboratory chemicals were of an analytically pure grade without further purification. Potassium hydroxide (KOH), ethanol anhydrous (C_2_H_5_OH), and manganese(II) chloride tetrahydrate (MnCl_2_·4H_2_O) were supplied by Guangdong Guanghua Technology Co., Ltd. (Guangdong, China) Nickel nitrate hexahydrate (Ni(NO_3_)_2_·6H_2_O) and methyl pyrrolidone (NMP) were provided by Tianjin Da Mao Chemical Reagent Factory and the Sinopharm Chemical Reagent Co., Ltd. (Tianjin, China) Ammonium fluoride (NH_4_F) delivered urea (CH_4_N_2_O) was purchased from Xi Long Science Co., Ltd. Ammonium heptamolybdate ((NH_4_)_6_Mo_7_O_24_·4H_2_O) was provided by Tianjin No. 4 Chemical Reagent Factory. Acetone (CH_3_COCH_3_) was supplied by Chengdu Ji Yuan Chemical Co., Ltd. (Chengdu, China) Kochen Black and polyvinylidene fluoride (PVDF900) were purchased from Guangdong Candlelight New Energy Co., Ltd. (Guangdong, China) Ni foam was obtained from Klute Chemical Co., Ltd. (Shanghai, China) Ruthenium oxide (RuO_2_) was purchased by Shanghai Aladdin Technology Co., Ltd. (Shanghai, China) Deionized water was manufactured by a laboratory ultrapure water machine.

### 2.2. Preparation of Precursors NiMoO_4_/NF

Prior to the synthesis, nickel foam was sonicated in 3 M HCl, ethanol, acetone, and deionized water for 15 min to remove the oxide layer and then vacuum dried. The NiMoO_4_ micro-rod array structure precursors were prepared by the conventional hydrothermal method [39]. Samples of 2.4 mmol of Ni(NO_3_)_2_·6H_2_O and 0.6 mmol of (NH_4_)_6_Mo_7_O_24_·4H_2_O were dissolved in 35 mL of deionized water and magnetically stirred for 30 min until the solution turned light green, after which the above resolution was transferred to a reaction vessel with a 50 mL polytetrafluoroethylene (PTFE) liner, then the pretreated nickel foam was added and held at 150 °C for 6 h. When left to cool naturally, the precursor was rinsed from the nickel foam with deionized and ethanolic solutions. Then, the NiMoO_4_·xH_2_O/NF precursor was placed in a high-temperature tube furnace and heated to 350 °C for 2 h under an argon atmosphere at a heating rate of 5 °C/min. The sample obtained was recorded as NiMoO_4_/NF.

### 2.3. Preparation of Mn-Doped-NiMoO_4_/NF

We precisely weighed 2.5 mmol of urea, 1 mmol of ammonium fluoride, and x mmol (x = 0.2, 0.25, 0.33) MnCl_2_·4H_2_O, keeping the molar ratio of Ni to Mn at (1:15, 1:12, 1:9), and 35 mL of deionized water, which was stirred magnetically for 20 min, and subsequently transferred to a reaction vessel with 50 mL with a PTFE liner, then the NiMoO_4_/NF precursor prepared in Section 2.2 above was added and reacted at 120 °C for 5 h. After being left to naturally cool, the sample was removed and then placed into a vacuum drying oven and dried at 60 °C for 12 h. These samples were recorded as Mn-doped-NiMoO_4_/NF(x) (x = 20, 25, 33 for 1:15, 1:12, 1:9, respectively). To better compare the effect of Mn ion doping on the electrocatalytic performance of the samples, Mn-doped-NiMoO_4_/NF (0) was prepared by the same process, except for the addition of manganese chloride.

### 2.4. Preparation of RuO_2_/NF

To better compare the activity of the catalysts, 16 mg of RuO_2_ powder, 2 mg of carbon black, 2 mg of PVDF, and 5 μL of NMP were added to an agate mortar and ground for about 15 min, during which ethanol was added as a dispersant as required. The prepared-ink was evenly applied to the ready Ni foam and subsequently dried under vacuum at 60 °C for 12 h to obtain RuO_2_/NF. After weighing, the RuO_2_ loading mass was approximately 5 mg cm^−2^.

### 2.5. Characterization

The XRD characterization in this work was carried out using a Smartlab model X-ray diffractometer manufactured by Rigaku, Japan. The experimental parameters used in the experiments were as follows: radiation type Cu Kα (λ = 0.15406 nm), voltage 40 kV, current 100 mA, scanning speed 8°/min, scanning range 5–70°. Raman characterization was through a Renishaw inVia Reflex laser Raman spectrometer. The laser power was 6 mW and the laser excitation wavelength was 532 nm. The scanning electron microscope (SEM) was a Sigma 300 field emission scanning electron microscope from Zeiss, Germany. In addition, the SEM was equipped with an X-ray energy spectrometer (EDS) to obtain the elemental surface distribution and the elemental content of the sample surface under the accelerating voltage of 20 kV. The microstructure of the material and the lattice stripes were obtained through a transmission electron microscope (TEM) Tecanai G2 F30 from FEI, USA, and the X-ray photoelectron spectroscopy (XPS) characterization was carried out using an ESCALAB 250XI from Thermo Fisher, USA. The ray type was Al Kα rays with a step size of 1 eV and a spot size of 650 μm, and the sample-making process was similar to SEM.

### 2.6. Electrochemical Measurement

All electrochemical tests in this work were carried out on an electrochemical workstation (CHI 660E Shanghai Chenhua). A standard three-electrode system was used: a Hg/HgO electrode as the reference electrode, graphite carbon rods as the counter electrode, and the experimentally prepared self-supporting material as the working electrode, using an electrolyte with a concentration of 1 M KOH. All tests were carried out in a constant temperature laboratory under 25 °C and the electrolyte needed to be saturated with O_2_ for 30 min before the OER tests were carried out. As a result, the potential in all tests will be converted to a reversible hydrogen electrode potential (RHE) E(RHE) = E(Hg/HgO) + 0.059 × pH + 0.098 (pH = 14). In addition, the scan rate of the linear scanning voltammetry (LSV) was set to a uniform 5 mV s^−1^ and the voltage range of the tests was 0 to 1 V. To accurately evaluate the overpotential in the OER tests, it was further corrected for iR compensation according to the formula E(RHE)_compensation_ = E(RHE) − iR. The cyclic voltammetry (CV) curves were recorded at sweep rates of 20–160 mV s^−1^ to calculate the double layer capacitance (CDL) of the electrode material and hence estimate the active electrochemical area (ECSA) of the electrocatalyst material. The electrochemical impedance spectroscopy (EIS) technique was used to test the frequency range of 0.1 to 100,000 Hz. The Nyquist diagram was fitted by Zview software (version: Zview2). The stability of the electrocatalyst materials was assessed by chrono-potentiometry (CP).

## 3. Results and Discussion

### 3.1. XRD and Raman Analysis

**Figure 1:**
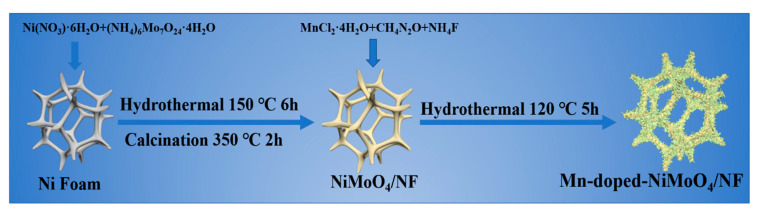
Schematic diagram of the synthesis process of Mn-doped-NiMoO_4_/NF.

Figure 1 shows the preparation of a Mn-doped-NiMoO_4_/NF self-supporting electrode. First, NiMoO_4_/NF micron-rod arrays were grown on nickel foam by the hydrothermal reaction and annealing calcination. Next, secondary hydrothermal doping of Mn elements and etching of the original micrometer rods into nanosheets to form a hierarchical structure were carried out. After a two-step hydrothermal reaction and one-step calcination, the color of the Ni foam changed from the original silver-grey into light green.

**Figure 2:**
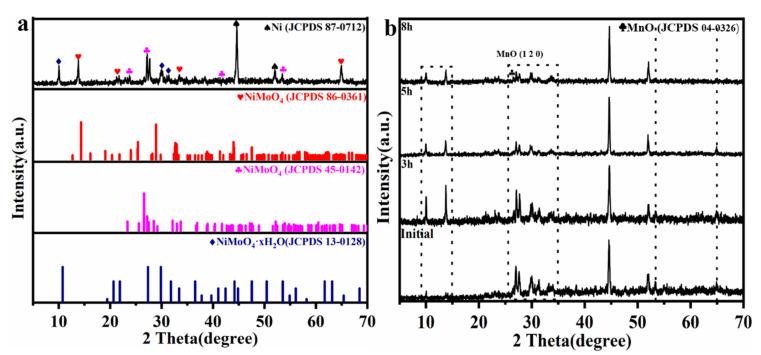
XRD patterns of (**a**) the precursors NiMoO_4_/NF and (**b**) Mn-doped-NiMoO_4_/NF with different reaction times (3 h, 5 h, 8 h).

The physical phase composition and crystal structure of NiMoO_4_/NF precursors and Mn-doped-NiMoO_4_/NF were studied by XRD, as shown in Figure 2a,b. Except for the diffraction peaks at 44.5°, 51.8°, and 76.4°, which perfectly matched the standard PDF card of metallic nickel foam (JCPDS 04-0850), the other diffraction peaks perfectly matched the standard PDF cards of NiMoO_4_·xH_2_O (JCPDS 13-0128) and NiMoO_4_ (JCPDS 86-0361 and JCPDS 45-0142) (as shown in Figure 2a) [40]. There was a significant decrease in the lattice parameters of the NiMoO_4_/NF precursors with the doping of Mn as the reaction time increased from 3 h to 8 h, as shown in Figure 2b. The diffraction peak was slightly shifted to the right, which can be attributed to the fact that the radius of the Mn^2+^ ion is smaller than that of the Ni^2+^ ion. In addition, a diffraction peak attributed to MnO (JCPDS 04-0326) could be observed around 26.6°, corresponding to a crystal plane of (1 2 0).

### 3.2. Raman Analysis

**Figure 3:**
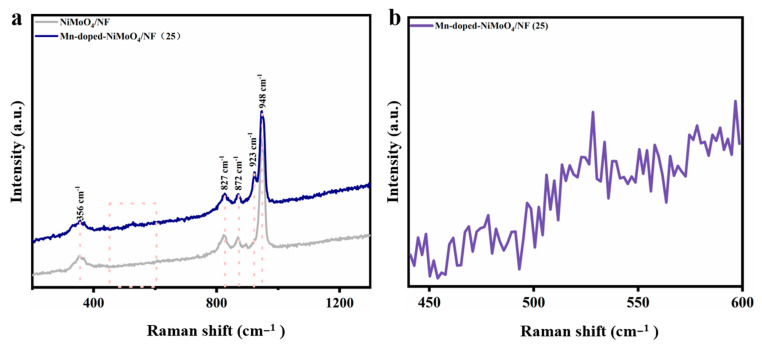
Raman spectra of (**a**) the Mn-doped-NiMoO_4_/NF (25) and NiMoO_4_/NF. (**b**) Enlarged view in the Raman shift range 440 cm^−1^–600 cm^−1^ of the Mn-doped-NiMoO_4_/NF (25).

To gain insights into the structural composition of the material, the electrocatalyst was further characterized by Raman spectroscopy. As shown in Figure 3a, the Mn-doped-NiMoO_4_/NF possessed characteristic peaks attributed to NiMoO_4_ at positions 356 cm^−2^, 827 cm^−2^, 872 cm^−2^, and 948 cm^−2^ [41]. The band at 923 cm^−1^ and 872 cm^−1^ indicates the symmetric stretching and asymmetric stretching bonds of the Mo–O bonds, respectively. Another band at 356 cm^−1^ was assigned to the bending vibration of the O–Mo–O units [42,43]. In contrast to NiMoO_4_/NF, the broad Raman band located in the range of 440 cm^−2^ to 600 cm^−2^ may be attributed to the Mn–O bonding vibrational mode of the oxides of amorphous Mn (Figure 3b). The above conclusions are consistent with the XRD results.

### 3.3. SEM and TEM Analysis

**Figure 4:**
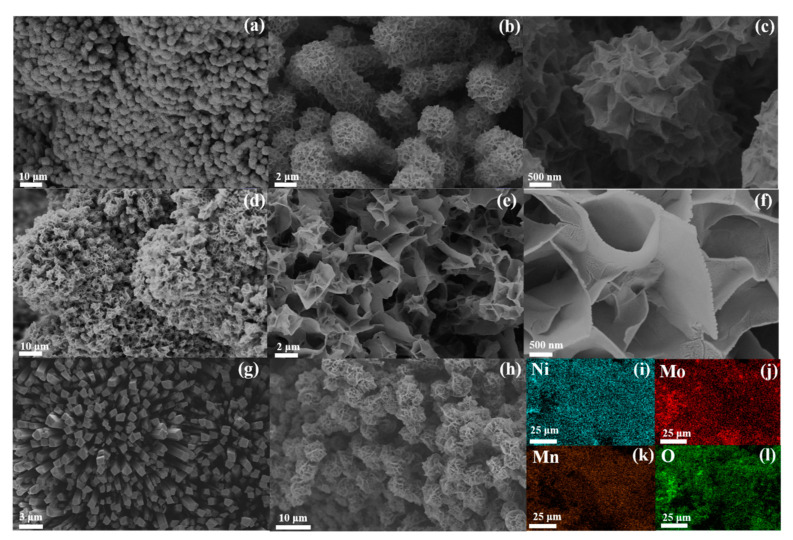
SEM images of (**a**–**c**) the Mn-doped-NiMoO_4_/NF (0), (**d**–**f**,**h**) the Mn-doped-NiMoO_4_/NF (25), (**g**) NiMoO_4_/NF/(**i**–**l**) EDS mapping images of the Mn-doped-NiMoO_4_/NF (25).

The microscopic morphology and elemental distribution of the material were characterized by SEM and an accompanying EDS spectrometer. Chemical etching formed many self-assembled nanosheets, which showed a uniform thickness of about 120 nm and a uniform distribution. Even with the high density of the nanosheets, the original rod-like array structure grown on nickel foam could still be vaguely distinguished, as shown in Figure 4h. By comparing (Figure 4a–c) Mn-doped-NiMoO_4_/NF (0) and (Figure 4d–f) Mn-doped-NiMoO_4_/NF (25), it can be seen that the size and the density of the nanosheets increased significantly after doping with Mn elements, and the gap in the nanosheets increased, which was conducive to further penetration of the electrolyte solution into the catalyst and accelerated the diffusion of bubbles. This suggests that the Mn element can modulate the morphology of the electrocatalyst material, providing more nanosheets and thus exposing more active sites and increasing the electrochemically active area, which may facilitate enhanced electrochemical activity. As shown in Supplementary Figure S1c,f,i, it was also observed that as the reaction time changed from 3 h to 8 h, the size of the nanosheets gradually became more significant. The different sized pores in the nanosheets accelerate the rate of electron transport and provide more channels for the release of oxygen [44]. As shown in Figure 4i–l, the Ni, Mn, Mo, and O elements were uniformly distributed in the Mn-doped-NiMoO_4_/NF (25).

**Figure 5:**
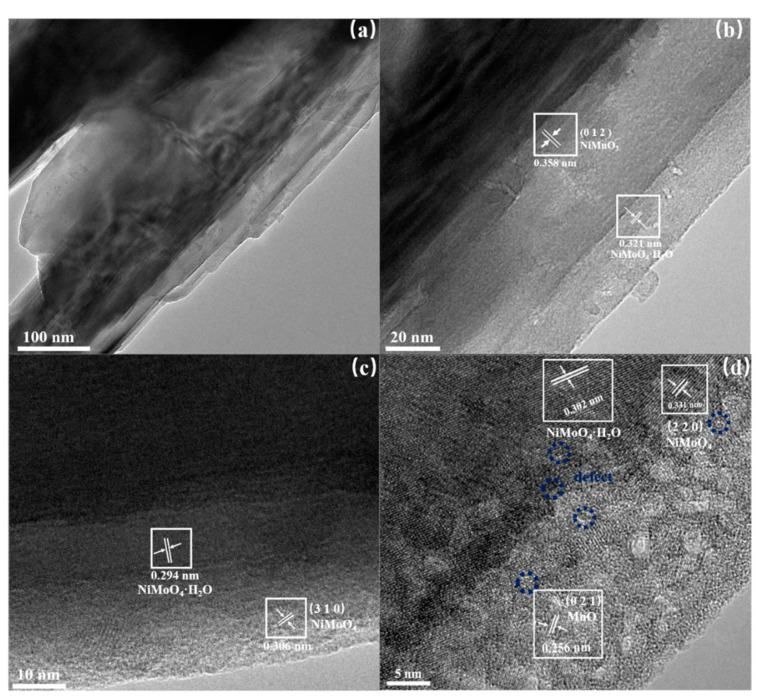
TEM images of (**a**–**d**) the Mn-doped-NiMoO_4_/NF (25).

The structure of the Mn-doped-NiMoO_4_ was further investigated by TEM. To avoid the influence of the nickel foam substrate on the test, a small sample was chosen for sonication in an ethanol solution, after which a pipette was used to measure and disperse a uniform drop onto the copper grid. As shown in Figure 5a, the rod-like structure of the electrocatalyst material could be observed, which is consistent with the characterization results of SEM. Therefore, a rod-like structure with a high aspect ratio can effectively increase the contact area between the catalyst and the electrolyte. Figure 5b–d further reveals that the Mn-doped-NiMoO_4_/NF (25) lattice stripe characteristics, as shown in Figure 5b, showed two different widths in the lattice stripe. The lattice stripe spacing was equal to 0.321 nm, corresponding to NiMoO_4_·H_2_O. In comparison, a small amount of Mn ions took the place of Mo ions to form NiMnO_3_ (JCPDS 48-1330), with a lattice stripe spacing of 0.358 nm corresponding to the crystal plane (0 1 2), as analyzed by the software Digital micrograph. As shown in Figure 5c,d, lattice stripes with a lattice spacing equal to 0.306 nm and 0.331 nm were observed that corresponded to the (3 1 0) and (2 2 0) crystal planes of NiMoO_4_ (JCPDS 86-0361 and JCPDS 45-0142), respectively. It could also be seen that there was a large amount of amorphous manganese oxide present on the surface of the material, with defects at the interface between the lattice stripe and the lattice stripe, favoring the exposure of more active sites [45].

### 3.4. XPS Analysis

**Figure 6:**
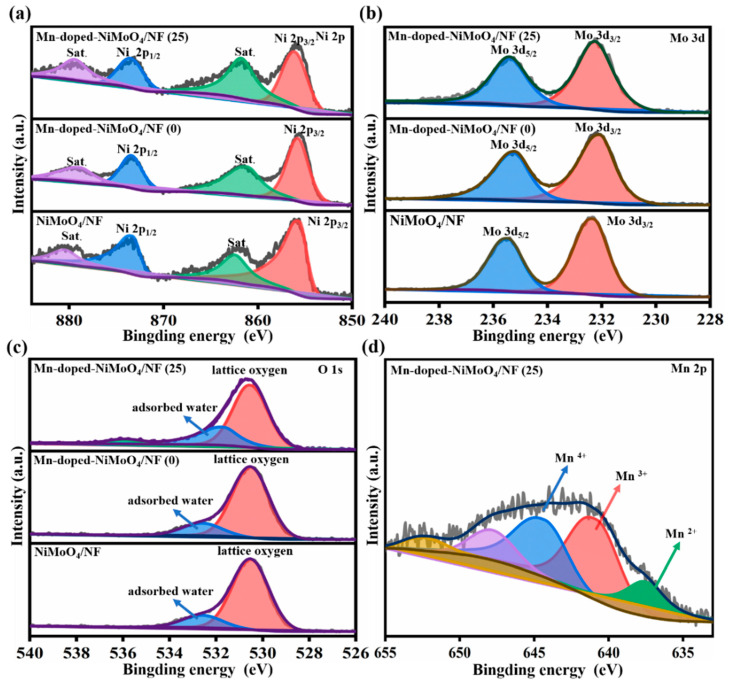
XPS analysis for the Mn-doped-NiMoO_4_/NF (x, x = 0, 25) and NiMoO_4_/NF in (**a**) Ni 2p, (**b**) Mo 3d, (**c**) O 1s, and (**d**) Mn 2p for Mn-doped-NiMoO_4_/NF (25).

The elemental composition and chemical states in the Mn-doped-NiMoO_4_/NF and NiMoO_4_/NF samples were investigated by XPS. The binding energy of the standard C 1s (284.80 eV) was used to correct the binding energy of all XPS spectra. The XPS spectra (Supplementary Figure S3a) show that Ni, Mo, Mn, O, and C co-existed in the Mn-doped-NiMoO_4_/NF (25), and the binding energies of the standard in Figure 6a shows that all of the spin-orbit splits of Ni 2p could be fitted to Ni 2p_3/2_ and Ni 2p_1/2_, accompanied by a satellite peak. Compared with Mn-doped-NiMoO_4_/NF (0) and NiMoO_4_/NF, the binding energy of Ni 2p_3/2_ (855.95 eV) in the Mn-doped-NiMoO_4_ /NF (25) shifted toward a higher binding energy with 0.23 eV and 0.32 eV; the binding energy of Ni 2p_1/2_ (873.51 eV) was also positively shifted with 0.12 eV and 0.21 eV; and the binding energy difference between Ni 2p_3/2_ and Ni 2p_1/2_ was 17.56 eV. The electron transfer in the nickel center may be attributed to the doping of the Mn elements and their interactions, suggesting that Ni^2+^ converted to a higher valance state [46]. As shown in Figure 6b, the binding energies at Mo 3d_5/2_ (231.86 eV) and Mo 3d_3/2_ (235.06 eV) shifted toward a lower binding energy with 0.37 eV and 0.39 eV compared to NiMoO_4_/NF. The peak separation of 3.2 eV indicates a high valence of Mo^6+^ species [47]. As shown in Figure 6c, the O 1s orbitals for Mn-doped-NiMoO_4_/NF (25) could be fitted to two peaks: lattice oxygen (O_1_: 530.53 eV), and absorbed oxygen may be attributed to surface oxygen defects/vacancy species (O_2_: 531.70 eV) [48]. The calculated S_O2_/(S_O2_ + S_O1_) for Mn-doped-NiMoO_4_/NF (25) was 30.2%, which was 10.1% higher than Mn-doped-NiMoO_4_/NF (0) and 13.4% higher than NiMoO_4_/NF, respectively, demonstrating that more oxygen defects are generated, which is beneficial to the overall electrocatalytic process [49]. The Mn 2p orbital for Mn-doped-NiMoO_4_/NF (25) could be fitted to three subpeaks at binding energies of 637.86 eV, 639.94 eV, and 642.56 eV, corresponding to Mn^2+^, Mn^3+^, and Mn^4+^, respectively (Figure 6d), while the other peaks were attributed to the oxidation state of the Mn formed on the surface. All of these results could help to confirm that Mn has been successfully doped into NiMoO_4_.

### 3.5. OER Performance Testing

**Figure 7:**
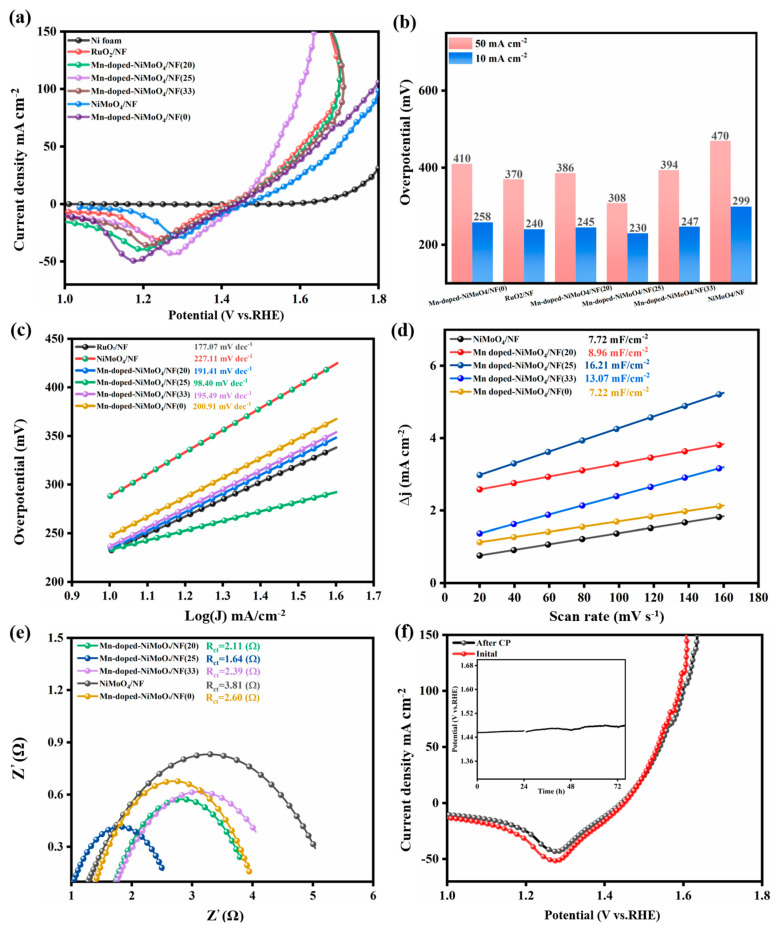
OER performance of the electrocatalyst samples. (**a**) LSV polarization curve. (**b**) Histogram of overpotentials for samples at 20 and 50 mA cm^−2^. (**c**) Tafel slope diagram. (**d**) The double-layer capacitance was obtained at different scan rates (20–160 mV s^−1^). (**e**) The EIS Nyquist plots at an open circuit voltage of 380 mV. (**f**) LSV polarization curves of Mn-doped-NiMoO_4_/NF (25) before and after 76 h of chronotropic potential testing at 10 mA cm^−2^.

In order to avoid the influence of the oxidation peak of Ni on the test, a reverse scan in the potential range 1–0 V was chosen to obtain a LSV polarization curve. To find the optimum reaction time, the electrocatalysts prepared at different reaction times (3 h, 5 h, and 8 h) were tested for LSV (Supplementary Figure S4), and the optimum reaction time was set at 5 h. The LSV curves of the different catalyst samples are shown in Figure 7a. The overpotential of the commercial RuO_2_/NF at 10 mA cm^−2^ was 242 mV. It is worth noting that Mn-doped-NiMoO4/NF (25) had the optimum overpotential (230 mV) when the current density was 10 mA cm^−2^, which was 62 mV lower than the pure NiMoO_4_/NF (299 mV). As shown in Figure 7b, Mn-doped-NiMoO_4_/NF (25) achieved the highest current density (225 mA cm^−2^) and exhibited a lower overpotential compared to other samples at both the 10 mA cm^−2^ and 50 mA cm^−2^ current densities of all samples in the potential range tested. These results indicate that the Mn-doped-NiMoO_4_/NF (25) catalyst had the optimum OER catalytic activity. The reason why the Mn-doped-NiMoO_4_/NF (25) sample showed excellent OER activity is that the content of Mn ions during the chemical etching process affects the surface reconstruction and enrichment of the high oxidation state hydroxide, and only the optimum amount of doping can further improve the OER catalytic activity of NiMoO_4_ [50,51].

The Tafel slope is an essential indicator of the kinetics of the catalytic reaction, with a smaller Tafel slope indicating a faster OER reaction as the potential increases [52]. As shown in Figure 7c, the Tafel slope of the control RuO_2_/NF was 177.07 mV dec^−1^, the Tafel slope of Mn-doped-NiMoO_4_/NF (25) was 98.40 mV dec^−1^ at higher current densities, while the Tafel slope of Mn-doped-NiMoO_4_/NF (0), Mn-doped-NiMoO_4_/NF (20), and Mn-doped-NiMoO_4_/NF (33) showed 200.91 mV dec^−1^, 191.41 mV dec^−1^, and 195.49 mV dec^−1^, respectively. This comparison shows that Mn-doped-NiMoO_4_/NF (25) had the fastest electrocatalytic OER kinetics.

To better explain the Mn-doped-NiMoO_4_/NF (25) OER performance, the electrochemical active surface area (ECSA) of different samples was evaluated by performing CV tests to calculate the C_dl_ of the material, and larger ECSA indicates more active sites exposed by the electrocatalyst. As shown in Figure 7d, the calculated C_dl_ values for NiMoO_4_/NF, Mn-doped-NiMoO_4_/NF (x) (x = 0, 20, 25, 33) were 7.22 mF/cm^−2^, 7.72 mF/cm^−2^, 8.96 mF/cm^−2^, 13.07 mF/cm^−2^, and 16.21 mF/cm^−2^, respectively. This result shows that the Mn-doped-NiMoO_4_/NF (25) catalyst material had the highest C_dl_ value, which proves that this catalyst material had the largest catalytically active surface area and exhibited the best electrocatalytic performance.

To further investigate the reaction kinetics on the catalyst surface, the electrochemical impedance spectra of the different catalyst samples were tested at an open circuit voltage of 0.38 V. The results are shown in Figure 7e. It can be seen that Mn-doped-NiMoO_4_/NF (25) had a minor semicircular diameter, which had an R_ct_ of 1.64 Ω. The other samples, NiMoO_4_/NF, Mn-doped-NiMoO_4_/NF (0), Mn-doped-NiMoO_4_/NF (20), and Mn-doped-NiMoO_4_/NF (33), had R_ct_ values of 3.81 Ω, 2.60 Ω, 2.11 Ω, and 2.39 Ω, respectively. This result indicates that Mn-doped-NiMoO_4_/NF (25) had faster reaction kinetics in the electrocatalytic OER process, which may be attributed to the coupling between Ni and Mn-doped metal oxides that enhances the charge transfer rate.

The stability of the catalyst is an important criterion to assess whether the catalyst can be used in the long-term. As shown in Figure 7f, the stability of Mn-doped-NiMoO_4_/NF (25) was tested for 76 h at a constant current density of 10 mA cm^−2^ using the chronopotential (CP), which showed an increase in overpotential of only 18 mV. The LSV polarization curves were compared before and after the CP test, and the difference between the two curves was not significant, demonstrating the excellent stability of the catalyst under alkaline conditions. After 76 h of chronopotential testing, the morphology of the nanosheets did not change significantly, but the gap between the rod arrays became slightly larger and the thickness of each nanosheet increased by nearly 20 nm (details can be found in Supplementary Figure S2). The main reasons for its long-term stability are mainly the rougher surface of the nanosheets and the wider gap in the rod array structure, which increases the direct contact area between the electrolyte and the electrocatalyst, thus exposing more active sites. As a side note, the appropriate density of electrocatalytic material optimizes the entire OER reaction process. According to the EDS energy spectrum analysis, the elements Ni, O, and Mn were still present on the surface of the electrocatalyst, but the amount of Mo was barely detectable. Raman testing of the sample after the chronopotentiometric method was carried out and revealed distinct Raman peaks at 477 cm^−1^ and 548 cm^−1^, attributed to the E_g_ (Ni^3+^–O) bending vibration mode and A_1g_(Ni^3+^–O) stretching vibration mode of γ-NiOOH, respectively [53] (details can be found in Supplementary Figure S6).

## 4. Conclusions

In summary, we successfully prepared self-supported electrodes (Mn-doped-NiMoO_4_/NF (25)) on 3D conductive nickel foam with a rod-like nanosheet array structure by a two-step hydrothermal and low-temperature calcination method. The electrocatalytic performance tests demonstrates that the doping of Mn elements could effectively modulate the NiMoO_4_/NF morphology, and the electronic valence state of its surface improved the electrical conductivity as well as provided more active sites. In addition, the tight connection between NiMoO_4_ and Ni foam effectively prevented agglomeration between the electrocatalysts, giving the catalyst excellent stability. In particular, the Mn-doped-NiMoO_4_/NF (25) only required 230 mV to drive a current density of 10 mA cm^−2^, and stable tests were completed for at least 76 h under alkaline conditions. During this process, the oxidation state of Ni increased and there was more Ni^3+^ as well as the rapid dissolution of MoO_4_^2−^, thus showing excellent OER activity. The designed electrocatalyst has the advantages of high activity and stability, and show the potential to be applied to the OER on a large scale. This work provides a new idea for the design and development of non-precious metal catalysts with a transition metal element doping strategy.

## Data Availability

Not applicable.

## Supplementary Materials

The following supporting information can be downloaded at: https://www.mdpi.com/article/10.3390/nano13050827/s1, Figure S1: SEM images of the Mn-doped-NiMoO4/NF (25) for different reaction times (3h, a–c; 5 h, d–f; 8 h, g–i); Figure S2: SEM images of (a) the Mn-doped-NiMoO_4_/NF (25) after 76 h CP test, (b–e) EDS mapping images of the Mn-doped-NiMoO_4_/NF (25) after 76 h CP test; Figure S3: XPS Survey of (a) Mn-doped-NiMoO_4_ /NF (25) and after CP: Mn-doped-NiMoO_4_/NF (25), (b) Mn 2p for after CP: Mn-doped-NiMoO_4_/NF; Figure S4: Different reaction times for the Mn-doped-NiMoO_4_/NF (25); Figure S5: CV plots for different samples at sweep speeds (20–160 mV s^−1^): (a) NiMoO_4_/NF (a), (b) Mn-doped-NiMoO_4_/NF (20), (c) Mn-doped-NiMoO_4_/NF (25), (d) Mn-doped-NiMoO_4_/NF (33), (e) Mn-doped-NiMoO_4_/NF (0); Figure S6: Raman spectra of after CP: Mn-doped-NiMoO_4_/NF (25); Table S1: Comparisons of OER activity for the Mn-doped electrocatalysts and NiMoO_4_-based electrocatalysts in alkaline conditions (η: overpotential at the current density of 10 mA cm^−2^). References [16,32,54,55,56,57,58,59,60] are mentioned in the Supplementary Materials.

## References

[1] Wu Y., Li F., Chen W., Xiang Q., Ma Y., Zhu H., Tao P., Song C., Shang W., Deng T. (2018). Coupling Interface Constructions of MoS_2_/Fe_5_Ni_4_S_8_ Heterostructures for Efficient Electrochemical Water Splitting. Adv. Mater..

[2] Seh Z.W., Kibsgaard J., Dickens C.F., Chorkendorff I., Norskov J.K., Jaramillo T.F. (2017). Combining theory and experiment in electrocatalysis: Insights into materials design. Science.

[3] Luo Y., Zhang Z., Chhowalla M., Liu B. (2022). Recent Advances in Design of Electrocatalysts for High-Current-Density Water Splitting. Adv. Mater..

[4] Kim J.S., Kim B., Kim H., Kang K. (2018). Recent Progress on Multimetal Oxide Catalysts for the Oxygen Evolution Reaction. Adv. Energy Mater..

[5] Zhao S., Yang Y., Tang Z. (2022). Insight into Structural Evolution, Active Sites, and Stability of Heterogeneous Electrocatalysts. Angew. Chem. Int. Ed. Engl..

[6] Li X., Kou Z., Xi S., Zang W., Yang T., Zhang L., Wang J. (2020). Porous NiCo_2_S4/FeOOH nanowire arrays with rich sulfide/hydroxide interfaces enable high OER activity. Nano Energy.

[7] Wang J., Wang J., Zhang M., Li S., Liu R., Li Z. (2020). Metal-organic frameworks-derived hollow-structured iron-cobalt bimetallic phosphide electrocatalysts for efficient oxygen evolution reaction. J. Alloys Compd..

[8] Yuan H., Wang S., Ma Z., Kundu M., Tang B., Li J., Wang X. (2021). Oxygen vacancies engineered self-supported B doped Co_3_O_4_ nanowires as an efficient multifunctional catalyst for electrochemical water splitting and hydrolysis of sodium borohydride. Chem. Eng. J..

[9] Lu J., Wang H., Sun Y., Wang X., Song X., Wang R. (2021). Charge state manipulation induced through cation intercalation into MnO_2_ sheet arrays for efficient water splitting. Chem. Eng. J..

[10] Ye Z., Li T., Ma G., Dong Y., Zhou X. (2017). Metal-Ion (Fe, V, Co, and Ni)-Doped MnO_2_ Ultrathin Nanosheets Supported on Carbon Fiber Paper for the Oxygen Evolution Reaction. Adv. Funct. Mater..

[11] Wang L., Wang J., Wang M., Li P., Tong J., Yu F. (2020). AgO-decorated multi-dimensional chrysanthemum-like NiCo_2_O_4_ mounted on nickel foam as a highly efficient and stable electrocatalyst for the oxygen evolution reaction. Nanoscale.

[12] Cheng C., Li D., Zhao T., Wang D., Zhong D., Hao G., Liu G., Li J., Zhao Q. (2021). NiFe_2_O_4_–Ni_3_S_2_ nanorod array/Ni foam composite catalyst indirectly controlled by Fe^3+^ immersion for an efficient oxygen evolution reaction. Int. J. Hydrogen Energy.

[13] Ji Y., Yang L., Ren X., Cui G., Xiong X., Sun X. (2018). Full Water Splitting Electrocatalyzed by NiWO_4_ Nanowire Array. ACS Sustain. Chem. Eng..

[14] Tian H., Zhang K., Feng X., Chen J., Lou Y. (2022). Self-supported CoMoO_4_/NiFe-LDH core-shell nanorods grown on nickel foam for enhanced electrocatalysis of oxygen evolution. Dalton Trans..

[15] Rajput A., Adak M.K., Chakraborty B. (2022). Intrinsic Lability of NiMoO_4_ to Excel the Oxygen Evolution Reaction. Inorg. Chem..

[16] An L., Feng J., Zhang Y., Wang R., Liu H., Wang G.-C., Cheng F., Xi P. (2019). Epitaxial Heterogeneous Interfaces on N-NiMoO_4_/NiS_2_ Nanowires/Nanosheets to Boost Hydrogen and Oxygen Production for Overall Water Splitting. Adv. Funct. Mater..

[17] Solomon G., Landström A., Mazzaro R., Jugovac M., Moras P., Cattaruzza E., Morandi V., Concina I., Vomiero A. (2021). NiMoO_4_@Co_3_O_4_ Core–Shell Nanorods: In Situ Catalyst Reconstruction toward High Efficiency Oxygen Evolution Reaction. Adv. Energy Mater..

[18] Wang F., Liu Z., Zhang K., Zha Q., Ni Y. (2021). Ce-Doped Ni-S nanosheets on Ni foam supported NiMoO_4_ micropillars: Fast electrodeposition, improved electrocatalytic activity and ultralong durability for the oxygen evolution reaction in various electrolytes. Dalton Trans..

[19] Zheng B., Fan J., Chen B., Qin X., Wang J., Wang F., Deng R., Liu X. (2022). Rare-Earth Doping in Nanostructured Inorganic Materials. Chem. Rev..

[20] Trotochaud L., Young S.L., Ranney J.K., Boettcher S.W. (2014). Nickel-Iron Oxyhydroxide Oxygen-Evolution Electrocatalysts: The Role of Intentional and Incidental Iron Incorporation. J. Am. Chem. Soc..

[21] Yin Z., Liang J., Xu H., Luo H., Deng D., Lu W., Long S. (2021). MoO_4_^2−^ doped Ni-Fe-Se nanospheres electrodeposited on nickel foam as effective electrocatalysts for oxygen evolution reaction. J. Electroanal. Chem..

[22] Tong Y., Chen P., Zhang M., Zhou T., Zhang L., Chu W., Wu C., Xie Y. (2017). Oxygen Vacancies Confined in Nickel Molybdenum Oxide Porous Nanosheets for Promoted Electrocatalytic Urea Oxidation. ACS Catal..

[23] Tian L., Li Z., Xu X., Zhang C. (2021). Advances in noble metal (Ru, Rh, and Ir) doping for boosting water splitting electrocatalysis. J. Mater. Chem. A.

[24] Luo L., Li W., Kang Y., Wang Z., Cheng X., Ruan M., Wu Q. (2022). Se and Fe co-doping in Ni_2_P/Ni1_2_P_5_/NF: Highly active and ultra-long stability of the oxygen evolution reaction. Appl. Surf. Sci..

[25] Tajik S., Askari M.B., Ahmadi S.A., Nejad F.G., Dourandish Z., Razavi R., Beitollahi H., Di Bartolomeo A. (2022). Electrochemical Sensor Based on ZnFe_2_O_4_/RGO Nanocomposite for Ultrasensitive Detection of Hydrazine in Real Samples. Nanomaterials.

[26] Askari M.B., Salarizadeh P., Di Bartolomeo A., Şen F. (2021). Enhanced electrochemical performance of MnNi_2_O_4_/rGO nanocomposite as pseudocapacitor electrode material and methanol electro-oxidation catalyst. Nanotechnology.

[27] Tang T., Jiang W.J., Niu S., Liu N., Luo H., Chen Y.Y., Jin S.F., Gao F., Wan L.J., Hu J.S. (2017). Electronic and Morphological Dual Modulation of Cobalt Carbonate Hydroxides by Mn Doping toward Highly Efficient and Stable Bifunctional Electrocatalysts for Overall Water Splitting. J. Am. Chem. Soc..

[28] Shi L., Fang H., Yang X., Xue J., Li C., Hou S., Hu C. (2021). Fe-cation Doping in NiSe_2_ as an Effective Method of Electronic Structure Modulation towards High-Performance Lithium-Sulfur Batteries. ChemSusChem.

[29] Zhu Y.P., Guo C., Zheng Y., Qiao S.Z. (2017). Surface and Interface Engineering of Noble-Metal-Free Electrocatalysts for Efficient Energy Conversion Processes. Acc. Chem. Res..

[30] Xu S., Yu X., Liu X., Teng C., Du Y., Wu Q. (2020). Contrallable synthesis of peony-like porous Mn-CoP nanorod electrocatalyst for highly efficient hydrogen evolution in acid and alkaline. J. Colloid Interface Sci..

[31] Xu S., Du Y., Liu X., Yu X., Teng C., Cheng X., Chen Y., Wu Q. (2020). Three-dimensional (3D) hierarchical coral-like Mn-doped Ni_2_P–Ni_5_P_4_/NF catalyst for efficient oxygen evolution. J. Alloys Compd..

[32] Duan J.J., Han Z., Zhang R.L., Feng J.J., Zhang L., Zhang Q.L., Wang A.J. (2021). Iron, manganese co-doped Ni_3_S_2_ nanoflowers in situ assembled by ultrathin nanosheets as a robust electrocatalyst for oxygen evolution reaction. J. Colloid Interface Sci..

[33] Sun H., Yan Z., Liu F., Xu W., Cheng F., Chen J. (2020). Self-Supported Transition-Metal-Based Electrocatalysts for Hydrogen and Oxygen Evolution. Adv. Mater..

[34] Li J., Lian R., Wang J., He S., Jiang S.P., Rui Z. (2020). Oxygen vacancy defects modulated electrocatalytic activity of iron-nickel layered double hydroxide on Ni foam as highly active electrodes for oxygen evolution reaction. Electrochim. Acta.

[35] Gong Y., Lin Y., Yang Z., Jiao F., Li J., Wang W. (2019). High-performance bifunctional flower-like Mn-doped Cu_7.2_S_4_@NiS_2_@NiS/NF catalyst for overall water splitting. Appl. Surf. Sci..

[36] Yu X., Xu S., Liu X., Cheng X., Du Y., Wu Q. (2021). Mn-doped NiCo_2_S_4_ nanosheet array as an efficient and durable electrocatalyst for oxygen evolution reaction. J. Alloys Compd..

[37] Jin C., Hou M., Li X., Liu D., Qu D., Dong Y., Xie Z., Zhang C. (2022). Rapid electrodeposition of Fe-doped nickel selenides on Ni foam as a bi-functional electrocatalyst for water splitting in alkaline solution. J. Electroanal. Chem..

[38] Liu S., Lv X., Liu G., Li C., Thummavichaia K., Li Z., Zhang L., Bin Z., Wang N., Zhu Y. (2022). In-situ fabrication of NixSey/MoSe_2_ hollow rod array for enhanced catalysts for efficient hydrogen evolution reaction. J. Colloid Interface Sci..

[39] Watcharatharapong T., Minakshi Sundaram M., Chakraborty S., Li D., Shafiullah G.M., Aughterson R.D., Ahuja R. (2017). Effect of Transition Metal Cations on Stability Enhancement for Molybdate-Based Hybrid Supercapacitor. ACS Appl. Mater. Interfaces.

[40] Zhuang S., Tong S., Wang H., Xiong H., Gong Y., Tang Y., Liu J., Chen Y., Wan P. (2019). The P/NiFe doped NiMoO_4_ micro-pillars arrays for highly active and durable hydrogen/oxygen evolution reaction towards overall water splitting. Int. J. Hydrogen Energy.

[41] Bankar P.K., Ratha S., More M.A., Late D.J., Rout C.S. (2017). Enhanced field emission performance of NiMoO_4_ nanosheets by tuning the phase. Appl. Surf. Sci..

[42] Chen Y.Y., Zhang Y., Zhang X., Tang T., Luo H., Niu S., Dai Z.H., Wan L.J., Hu J.S. (2017). Self-Templated Fabrication of MoNi_4_ /MoO_3-x_ Nanorod Arrays with Dual Active Components for Highly Efficient Hydrogen Evolution. Adv. Energy Mater..

[43] Ghosh D., Giri S., Das C.K. (2013). Synthesis, characterization and electrochemical performance of graphene decorated with 1D NiMoO_4_·nH_2_O nanorods. Nanoscale.

[44] Chen M.T., Duan J.J., Feng J.J., Mei L.P., Jiao Y., Zhang L., Wang A.J. (2022). Iron, rhodium-codoped Ni_2_P nanosheets arrays supported on nickel foam as an efficient bifunctional electrocatalyst for overall water splitting. J. Colloid Interface Sci..

[45] Zhang L., Chen Y., Liu G., Li Z., Liu S., Tiwari S.K., Ola O., Pang B., Wang N., Zhu Y. (2022). Construction of CoP/Co_2_P Coexisting Bifunctional Self-Supporting Electrocatalysts for High-Efficiency Oxygen Evolution and Hydrogen Evolution. ACS Omega.

[46] Gong Y., Yang Z., Zhi Y., Lin Y., Zhou T., Li J., Jiao F., Wang W. (2019). Controlled synthesis of bifunctional particle-like Mo/Mn-Ni_x_S_y_/NF electrocatalyst for highly efficient overall water splitting. Dalton Trans..

[47] Chen C.X., He S.Q., Dastafkan K., Zou Z.H., Wang Q.X., Zhao C.A. (2022). Sea urchin-like NiMoO_4_ nanorod arrays as highly efficient bifunctional catalysts for electrocatalytic/photovoltage-driven urea electrolysis. Chin. J. Catal..

[48] Xie C., Wang Y., Hu K., Tao L., Huang X., Huo J., Wang S. (2017). In situ confined synthesis of molybdenum oxide decorated nickel–iron alloy nanosheets from MoO_4_^2−^ intercalated layered double hydroxides for the oxygen evolution reaction. J. Mater. Chem. A.

[49] Hai G.J., Huang J.F., Cao L.Y., Kajiyoshi K., Wang L., Feng L.L. (2021). Hierarchical W_18_O_49_/NiWO_4_/NF heterojunction with tuned composition and charge transfer for efficient water splitting. Appl. Surf. Sci..

[50] Wang Y., Zhu Y., Zhao S., She S., Zhang F., Chen Y., Williams T., Gengenbach T., Zu L., Mao H. (2020). Anion Etching for Accessing Rapid and Deep Self-Reconstruction of Precatalysts for Water Oxidation. Matter.

[51] Zhu J., Qian J., Peng X., Xia B., Gao D. (2023). Etching-Induced Surface Reconstruction of NiMoO_4_ for Oxygen Evolution Reaction. Nano-Micro Lett..

[52] Suen N.T., Hung S.F., Quan Q., Zhang N., Xu Y.J., Chen H.M. (2017). Electrocatalysis for the oxygen evolution reaction: Recent development and future perspectives. Chem. Soc. Rev..

[53] Gou W., Chen Y., Zhong Y., Xue Q., Li J., Ma Y. (2022). Phytate-coordinated nickel foam with enriched NiOOH intermediates for 5-hydroxymethylfurfural electrooxidation. Chem. Commun..

[54] Xu H., Shang H., Di J., Du Y. (2019). Geometric and Electronic Engineering of Mn-Doped Cu(OH)_2_ Hexagonal Nanorings for Superior Oxygen Evolution Reaction Electrocatalysis. Inorg. Chem..

[55] Teng Y., Wang X.-D., Liao J.-F., Li W.-G., Chen H.-Y., Dong Y.-J., Kuang D.-B. (2018). Atomically Thin Defect-Rich Fe-Mn-O Hybrid Nanosheets as High Efficient Electrocatalyst for Water Oxidation. Adv. Funct. Mater..

[56] Wang Z., Wang H., Ji S., Wang X., Zhou P., Huo S., Linkov V., Wang R. (2020). A High Faraday Efficiency NiMoO_4_ Nanosheet Array Catalyst by Adjusting the Hydrophilicity for Overall Water Splitting. Chemistry.

[57] Padmanathan N., Shao H., Razeeb K.M. (2020). Honeycomb micro/nano-architecture of stable β-NiMoO_4_ electrode/catalyst for sustainable energy storage and conversion devices. Int. J. Hydrogen Energy.

[58] Ehsan M.A., Khan A. (2021). Aerosol-Assisted Chemical Vapor Deposition Growth of NiMoO_4_ Nanoflowers on Nickel Foam as Effective Electrocatalysts toward Water Oxidation. ACS Omega.

[59] Duan Y., Huang Z., Zhao C., Ren J., Dong X., Jia R., Xu X., Shi S. (2021). In-Situ Generated Trimetallic Molybdate Nanoflowers on Ni Foam Assisted with Microwave for Highly Enhanced Oxygen Evolution Reaction. Chemistry.

[60] Wang J., Hu J., Niu S., Li S., Du Y., Xu P. (2022). Crystalline-Amorphous Ni_2_P_4_O_12_ /NiMoOx Nanoarrays for Alkaline Water Electrolysis: Enhanced Catalytic Activity via In Situ Surface Reconstruction. Small.

