# Supplementary material for: In Situ Fabrication of Mn-Doped NiMoO4 Rod-like Arrays as High Performance OER Electrocatalyst
Source: Nanomaterials (Basel). 2023 Feb 23;13(5):827. doi: 10.3390/nano13050827 (PMC10005328; doi:10.3390/nano13050827)
Supplement: Supplementary file 1 [file nanomaterials-13-00827-s001.zip › nanomaterials-2213127-supplementary.pdf]

Supplementary Materials

In Situ Fabrication of Mn-Doped $NiMoO_4$ Rod-Like Arrays as High Performance OER Electrocatalyst

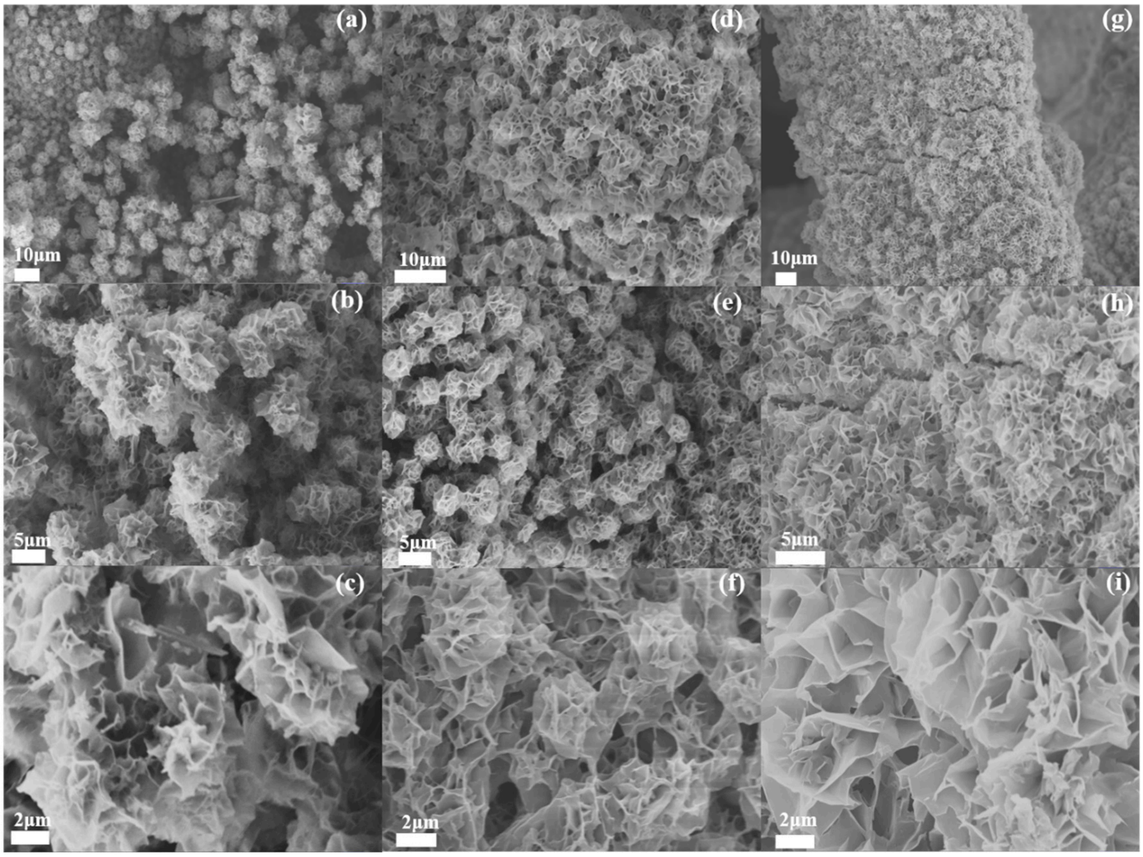

Figure S1. SEM images of the Mn-doped-$NiMoO_4$/NF (25) for different reaction times (3 h, a-c; 5 h, d-f; 8 h, g-i;).

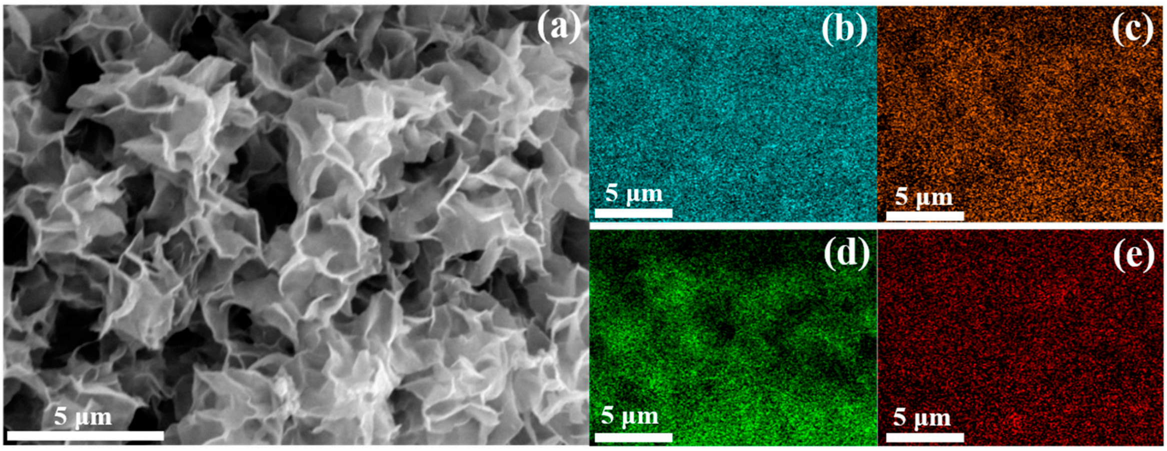

Figure S2. SEM images of (a) the Mn-doped-$NiMoO_4$/NF (25) after 76 h CP test, (b-e) EDS mapping images of the Mn-doped-$NiMoO_4$/NF (25) after 76 h CP test.

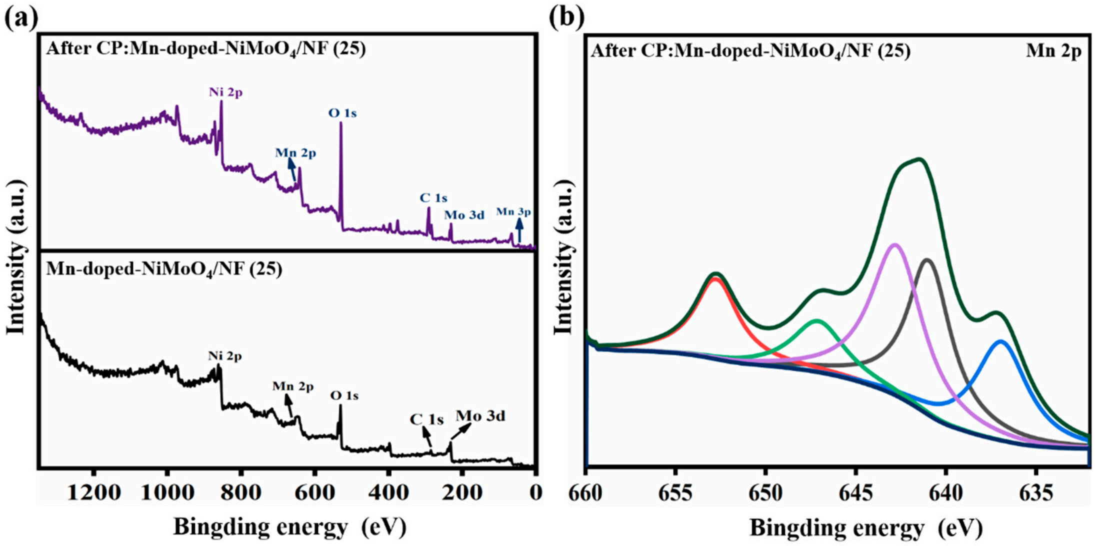

Figure S3. XPS Survey of (a) Mn-doped-$NiMoO_4$ /NF (25) and After CP:Mn-doped-$NiMoO_4$ /NF (25), (b) Mn 2p for After CP:Mn-doped-$NiMoO_4$/NF.

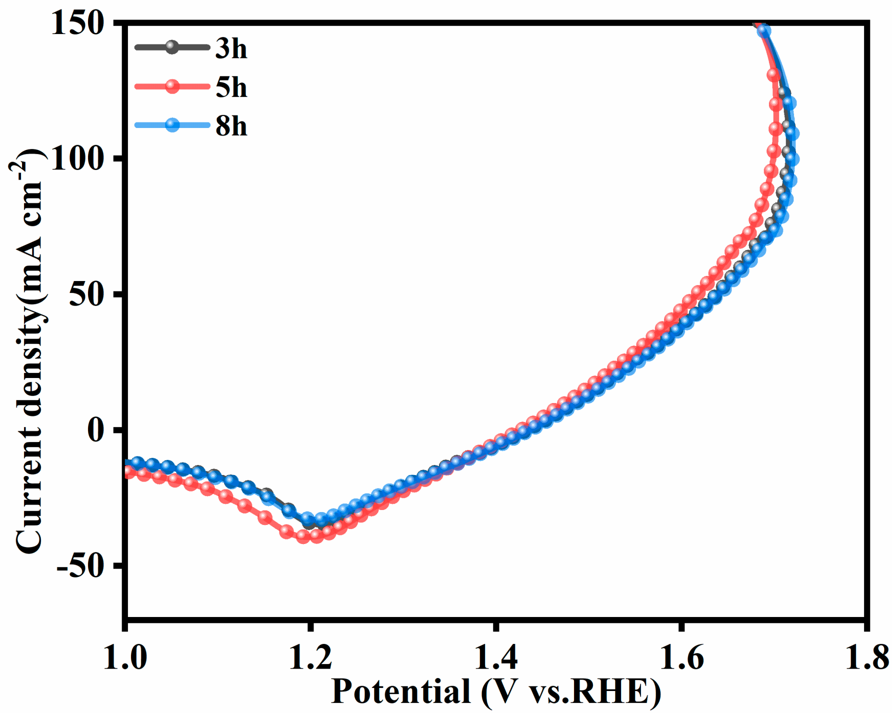

Figure S4. Different reaction times for the Mn-doped-$NiMoO_4$ /NF (25).

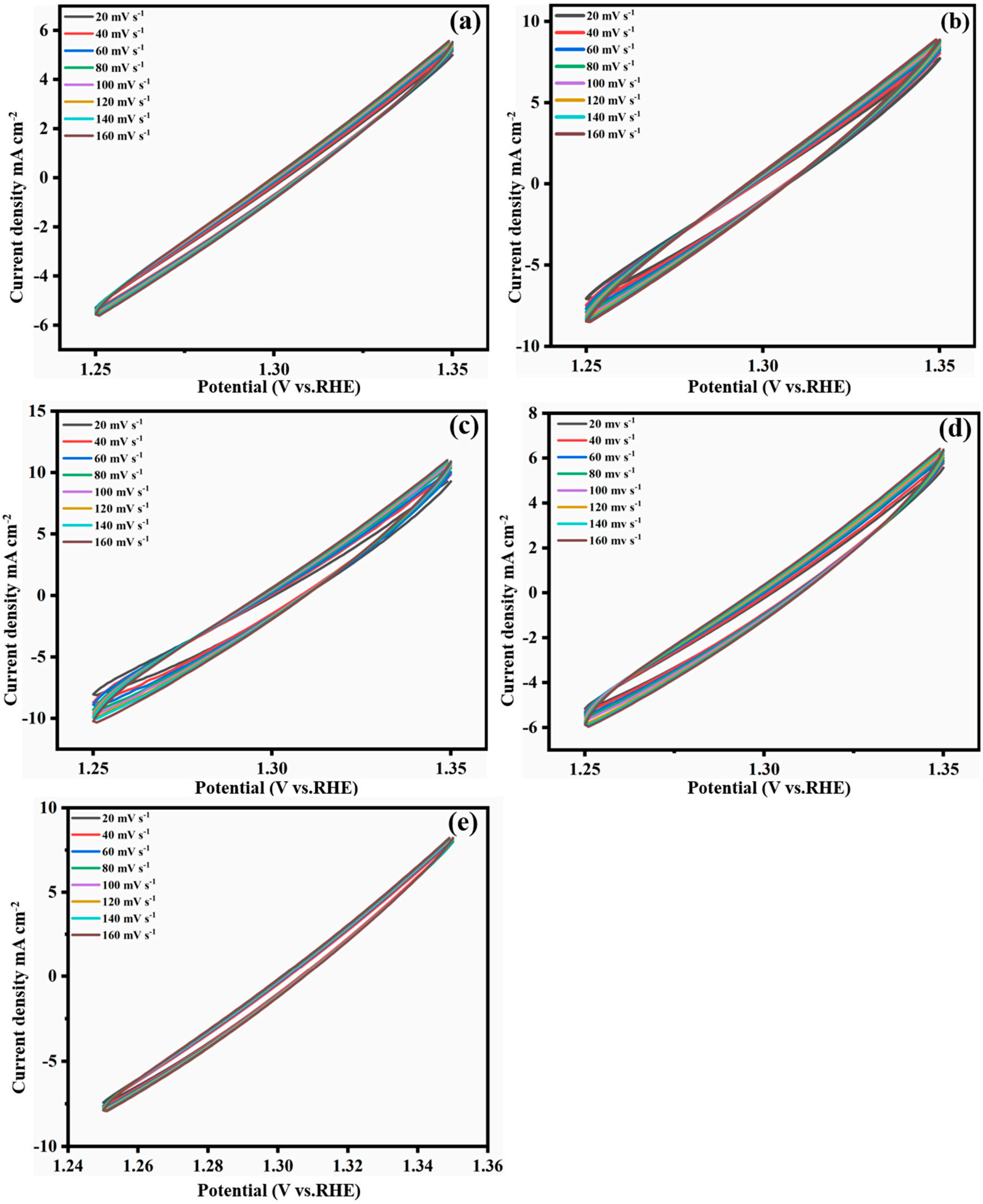

Figure S5. CV plots for different samples at sweep speeds (20-160 mV s^{-1}): (a) $NiMoO_4$/NF (a), (b) Mn-doped-$NiMoO_4$/NF (20), (c) Mn-doped-$NiMoO_4$/NF (25), (d) Mn-doped-$NiMoO_4$/NF (33), (e) Mn-doped-$NiMoO_4$/NF (0).

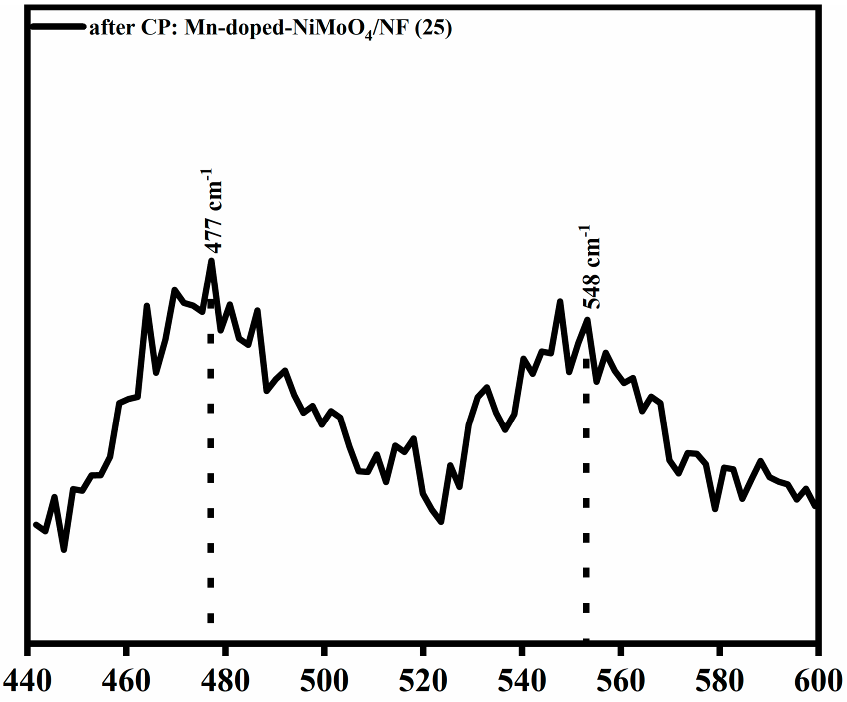

Figure S6. Raman spectra of after CP: Mn-doped-NiMoO4/NF (25).

Table S1. Comparisons of OER activity for Mn-doped electrocatalysts and $NiMoO_4$-based electrocatalysts in alkaline conditions (η: overpotential at the current density of 10 mA cm^{-2})

Catalyst	η(mV)	Electrolyte	Reference
**Mn-doped-$NiMoO_4$/NF (25)**	**230**	**1.0 M KOH**	**This work**
**Mn-doped $Cu(OH)_2$ HNs**	282	1.0 M KOH	1
**Fe-Mn-O NSs/CC**	273	1.0 M KOH	2
**Fe, Mn-Ni_3S_2/NF**	216	1.0 M KOH	3
**NM-FC_2**	336	1.0 M KOH	4
**N-$NiMoO_4$/NiS_2**	267	1.0 M KOH	5
**β- $NiMoO_4$/NF**	351	1.0 M KOH	6
**NM1**	320	1.0 M KOH	7
**$Ni_{0.9}Al_{0.1}MoO_4$/NF**	266	1.0 M KOH	8
**$Ni_2P_4O_{12}$ /NiMoOx**	250	1.0 M KOH	9

References

1. Xu, H.; Shang, H.; Di, J.; Du, Y., Geometric and Electronic Engineering of Mn-Doped $Cu(OH)_2$ Hexagonal Nanorings for Superior Oxygen Evolution Reaction Electrocatalysis. *Inorganic Chemistry* **2019,** *58* (22), 15433-15442.
2. Teng, Y.; Wang, X.-D.; Liao, J.-F.; Li, W.-G.; Chen, H.-Y.; Dong, Y.-J.; Kuang, D.-B., Atomically Thin Defect-Rich Fe-Mn-O Hybrid Nanosheets as High Efficient Electrocatalyst for Water Oxidation. *Advanced Functional Materials* **2018,** *28* (34), 1802463.
3. Duan, J. J.; Han, Z.; Zhang, R. L.; Feng, J. J.; Zhang, L.; Zhang, Q. L.; Wang, A. J., Iron, manganese co-doped Ni_3S_2 nanoflowers in situ assembled by ultrathin nanosheets as a robust electrocatalyst for oxygen evolution reaction. *Journal of Colloid and Interface Science* **2021,** *588*, 248-256.
4. Wang, Z.; Wang, H.; Ji, S.; Wang, X.; Zhou, P.; Huo, S.; Linkov, V.; Wang, R., A High Faraday Efficiency $NiMoO_4$ Nanosheet Array Catalyst by Adjusting the Hydrophilicity for Overall Water Splitting. *Chemistry* **2020,** *26* (52), 12067-12074.
5. An, L.; Feng, J.; Zhang, Y.; Wang, R.; Liu, H.; Wang, G.-C.; Cheng, F.; Xi, P., Epitaxial Heterogeneous Interfaces on N-$NiMoO_4$/NiS_2 Nanowires/Nanosheets to Boost Hydrogen and Oxygen Production for Overall Water Splitting. *Advanced Functional Materials* **2019,** *29* (1), 1805298.
6. Padmanathan, N.; Shao, H.; Razeeb, K. M., Honeycomb micro/nano-architecture of stable β-$NiMoO_4$ electrode/catalyst for sustainable energy storage and conversion devices. *International Journal of Hydrogen Energy* **2020,** *45* (55), 30911-30923.
7. Ehsan, M. A.; Khan, A., Aerosol-Assisted Chemical Vapor Deposition Growth of $NiMoO_4$ Nanoflowers on Nickel Foam as Effective Electrocatalysts toward Water Oxidation. *ACS Omega* **2021,** *6* (46), 31339-31347.
8. Duan, Y.; Huang, Z.; Zhao, C.; Ren, J.; Dong, X.; Jia, R.; Xu, X.; Shi, S., In-Situ Generated Trimetallic Molybdate Nanoflowers on Ni Foam Assisted with Microwave for Highly Enhanced Oxygen Evolution Reaction. *Chemistry* **2021,** *27* (35), 9044-9053.
9. Wang, J.; Hu, J.; Niu, S.; Li, S.; Du, Y.; Xu, P., Crystalline-Amorphous $Ni_2P_4O_{12}$ /NiMoOx Nanoarrays for Alkaline Water Electrolysis: Enhanced Catalytic Activity via In Situ Surface Reconstruction. *Small* **2022,** *18* (10), 2105972.
